# Dataset of next-generation sequence reads of nanobody clones in phage display library derived from Indian desert camel (*Camelus dromedarius* L.)

**DOI:** 10.1016/j.dib.2020.106663

**Published:** 2020-12-16

**Authors:** Somesh Banerjee, Ajit Singh, Jagveer Rawat, Nitish Bansal, Sushila Maan

**Affiliations:** a Immunology Section, Department of Veterinary Microbiology, India; b Department of Veterinary Public Helth & Epidemiology, India; c Department of Animal Biotechnology, Lala Lajpat Rai University of Veterinary and Animal Sciences, Hisar, Haryana 125004, India

**Keywords:** Dataset, NGS reads archive, Nanobodies, Phage display library, Indian desert camel

## Abstract

Next-generation sequences (NGS) dataset of nanobody (Nb) clones in a phage display library (PDL) is of immense value as it serves in many different ways, such as: i). estimating the library size, ii). improving selection and identification of Nbs, iii). informing about frequency of V gene families, diversity and length of CDRs, iv). high resolution analysis of natural and synthetic libraries, etc. [1], [2], [3]. We used a fraction of our previously constructed PDL of Nbs derived from an *E. coli* lipopolysaccharide-immunized Indian desert camel in order to obtain the dataset of NGS reads of Nbs. The cryo-preserved transformants library was revived to extract the Nb-encoding VHH (inserts)-pHEN4 (vector) DNA pool. The DNA sample was used for amplifying VHH pool by PCR [6]. The VHH amplicons band was gel-purified and subjected to NGS using Illumina MiSeq^TM^ platform. ‘Nextra XT micro V2 Index’ kit was used for the Nb library DNA sample sequencing, with the adaptors: ‘i7’ (N706: TAGGCATG) and ‘i5’ (S517: GCGTAAGA). The raw data comprised of a total read count of 182146 (matched= 179591; unmatched=2555), with average read length of 130.33 bases and a total of 23.74 Mb. Of 179591 matched reads, 142004 were paired reads and 37587 broken paired reads. The raw data of NGS reads was submitted to NCBI Sequence Reads Archive accessible at URL: https://www.ncbi.nlm.nih.gov/Traces/study/?acc=PRJNA516512 (dataset ref. [7]), and after analysis deposited in Mendeley Datasets repository, which is accessible at URL: [https://data.mendeley.com/datasets/4rsz3snvk5/3] (dataset ref. [8]). The sequence reads were analyzed by bioinformatics tools [9], [10], [11], [12]. The assembled consensus contigs revealed Nb orthologs of diverse Ag-specificities, including those isolated by conventional panning and Sanger-sequenced functional Nbs. Contig 1 CDR1-3 matched to those of anti-*Trypanosoma evansi* RoTat1.2 variant surface glycoprotein (VSG), while Contig 2 CDR1-3 matched to those of anti-LPS Nb clones isolated from the library. Contig 3 was however incomplete and lacked CDR3. Despite lacking the depth, the NGS data is a useful guide for selection of antigen-specific Nbs from the library, as demonstrated by anti-*T. evansi* VSG Nbs, and provides templates for Nb-based diagnostic reagents and therapeutic agents.

## Specifications Table

**Table:** 

Subject	Biological Sciences: Immunology
Specific subject area	Immunology: Domain antibodies or nanobodies library; phage display technology platform
Type of data	Table Image Graph Figure Raw data: Next-generation sequence reads linked to Mendeley Datasets Repository [https://data.mendeley.com/datasets/4rsz3snvk5/3] & NCBI Sequence Reads Archive [raw NGS data files only) [https://www.ncbi.nlm.nih.gov/Traces/study/?acc=PRJNA516512]
How data were acquired	The data acquired: Next-generation sequence reads raw data Instruments: Illumina®, Inc., USA Make and model and of the instruments used: MiSeq™ sequencing system. The data analysis: In-built MiSeq™ software and CLC Genomics Workbench 12.0 (Qiagen Bioinformatics) BLAST at: https://blast.ncbi.nlm.nih.gov/Blast.cgi (21 May 2019; 23 November 2020; 29 November 2020) IgBLAST at: https://www.ncbi.nlm.nih.gov/igblast/igblast.cgi (18 November 2020; 03 December 2020)
Data format	Raw Analysed Phylogenetic tree
Parameters for data collection	NGS sequence reads of the nanobody clones in the phage display library derived from an LPS-immunized Indian desert camel (*Camelus dromedarius* L.).
Description of data collection	Nanobody-encoding VHH (inserts)-pHEN4 (vector) DNA was extracted from *E. coli* host strain TG1 transformant library, and the VHH pool amplified by PCR. The gel-purified VHH-PCR amplicons were subjected to NGS using Illumina MiSeq^TM^ system. The NGS data comprising of paired-end reads of 150 bp length of the library DNA sample was acquired using ‘Nextra XT micro V2 Index’ kit, with following adaptors: ‘i7’ (N706: TAGGCATG) and ‘i5’ (S517: GCGTAAGA).
Data source location	Institution: Lala Lajpat Rai University of Veterinary and Animal Sciences, Hisar-125004 India City/Town/Region: Hisar, Haryana Country: India
Data accessibility	Repository name: [Mendeley Datasets Repository] Data identification number: [10.17632/4rsz3snvk5.3] Direct URL to data: [https://data.mendeley.com/datasets/4rsz3snvk5/3] Instructions for accessing these data: Accessible using the hyperlink URL: https://data.mendeley.com/datasets/4rsz3snvk5/3 Repository name: NCBI Sequence Reads Archive (SRA) database Direct URL to SRA data of PDL-NGS sequence reads: [https://www.ncbi.nlm.nih.gov/Traces/study/?acc=PRJNA516512] Direct URL to NCBI Blast: [https://blast.ncbi.nlm.nih.gov/Blast.cgi] [https://www.ncbi.nlm.nih.gov/igblast/igblast.cgi]
Related research article	P. Jangra, A. Singh, *Staphylococcus aureus* β-hemolysin-neutralizing single-domain antibody isolated from phage display library of Indian desert camel, *Asian Pac. J. Trop. Med.***3**(1) (2010) 1–7.
	https://doi.org/10.1016/S1995-7645(10)60020-X

## Value of the Data

• The NGS dataset is a resource of endotoxin-neutralizing and other biotechnologically and clinically useful nanobody clones derived for the first time from an immunized Indian desert camel.
• The investigators interested in the comparative studies on mammalian Ig-VH repertoire evolution, and those interested in novel nanobody-based drug designs will benefit the most.
• The dataset provides phylogenetically related templates for reformatting and re-designing of novel nanobody constructs.

## 1 Data Description

### 1.1 Dataset of NGS reads of Nbs in phage display library derived from LPS-immunized Indian desert camel

**Fig. 1:**
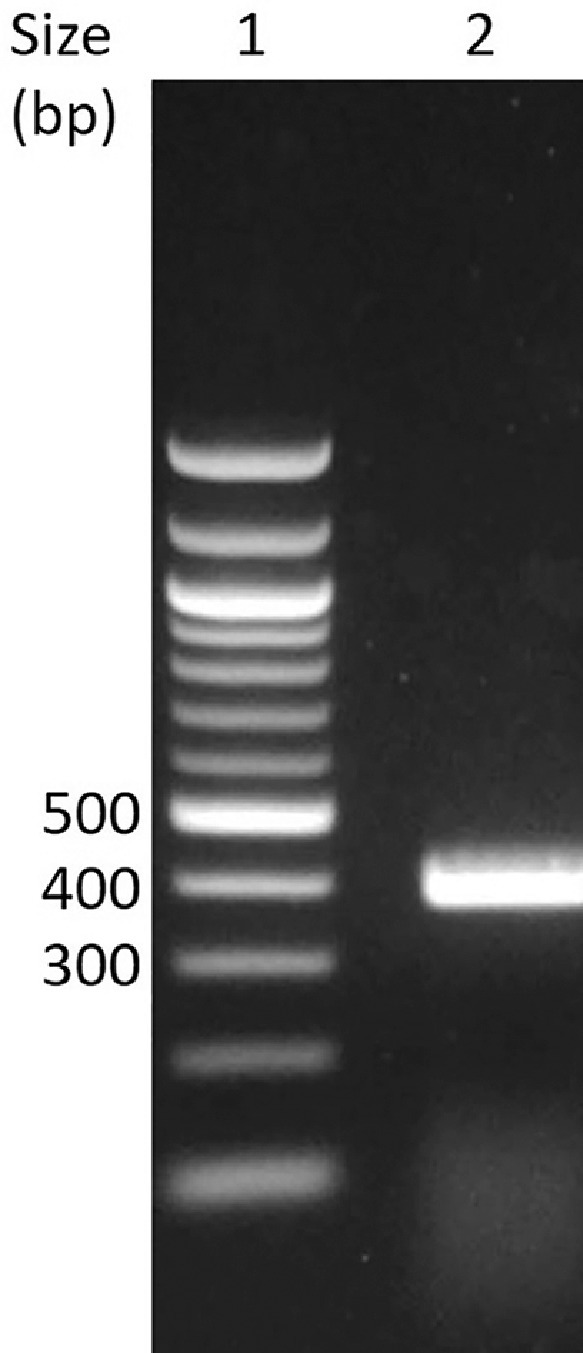
Agarose gel electrophoresis in 2% agarose in 1x tris-acetate-EDTA buffer showing PCR product of the nanobody library [Lane 1: 100 bp ladder of DNA size markers; 2: PCR amplicon of the nanobody gene library in the size range of 370–420 bp]

The cryo-preserved culture of TG1 transformant library was revived. The insert-vector (VHH-pHEN4) DNA extracted and purified from 100 mL of the transformants library culture was found to be 70 μg/mL. with A_260_/A_280_ ratio of 2.0. The library of nanobody-encoding VHH clones was amplified by PCR from the purified plasmid DNA and the PCR products purified from agarose gel. PCR products resolved by agarose gel electrophoresis (AGE) are shown in Fig. 1 as a thick band of about 400 bp, representing the VHH library. The concentration of the PCR products was found to be 230 ng/μL, with A_260_/A_280_ ratio of 2.1. The PCR amplified DNA was adjusted to 0.2 μg/μL buffer and 50 μL submitted for NGS by Illumina® MiSeq™ system.

Raw NGS data ‘Read 1’ [Phage lib NGS seqs-ABT-28_S24_L001_R1_001.fastq (1)] and ‘Read 2’ [Phage lib NGS seqs-ABT-28_S24_L001_R2_001.fastq (2)] files were generated by Illumina® MiSeq™ system. The raw NGS data mentioned above were deposited at NCBI Sequence Reads Archive (SRA) and the run SRR8477290 is accessible at URL: [https://www.ncbi.nlm.nih.gov/Traces/study/?acc=PRJNA516512] (dataset ref. [7]). Preliminary analysis was done and the NGS data files were submitted on December 05, 2020 to Mendeley Datasets Repository, and the published dataset can be accessed at URL: [https://data.mendeley.com/datasets/4rsz3snvk5/3] (dataset ref. [8]). Preliminary examination using CLC Genomics Workbench 12.0 (Qiagen Bioinformatics) revealed 91,073 sequence reads in each file (Mendeley dataset files [8]). A total read count was 182146 (matched= 179,591; unmatched=2555), with average read length of 130.33 bases and a total of 23.74 Mb. Of 179591 matched reads, 142004 were paired reads and 37,587 broken paired reads; 77,249 mapped and 13,824 not-mapped reads (Mendeley dataset files: ABT-28_S24_L001_R1_001_24 (paired) assembly summary report; ABT-28_S24_L001_R2_001 assembly coverage analysis report; ABT-28_S24_L001_R1_001 mapping summary report [8]). Quality score ‘Q30’ (1 in 1000 bases incorrect) at 2 × 150 bp was >80%, and overall QC report was acceptable (Mendeley dataset files: ABT-28_S24_L001_R2_001 - duplicated sequences QC report, ABT-28_S24_L001_R2_001 - supplementary QC report [8]).

### 1.2 Bioinformatics of NGS reads and contigs

**Fig. 2:**
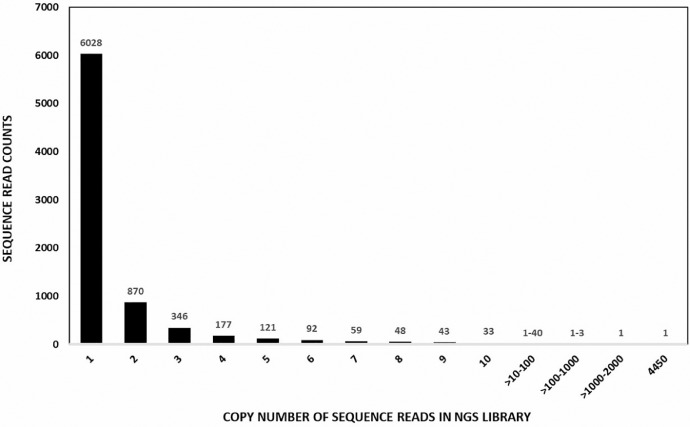
Copy number of sequence- reads in NGS data of phage display library.

De Novo Assembler (CLC Genomics Workbench 12.0) generated 3 contigs of 384 (total read count=155,238), 484 (total read count=24212) and 253 (total read count=103) bases as shown in Table 1 (Mendeley dataset files: Phage display library-NGS Contigs table; ABT-28_S24_L001_R1_001_24 (paired) assembly summary report [8]). The mapped consensus sequences of contig 1, -2 and -3 are presented in Table 2 (Mendeley dataset file: Phage display library-NGS Contigs table [8]). Gene duplication/copy number of reads in the present NGS data is shown in Fig. 2. About 71% of the sequences were unique (single copy), whereas one sequence had >4000 copy numbers and nine sequences had >1000 copy numbers.

**Table 1:** De Novo Assembly* (paired) of NGS reads of phage display library of Indian desert camel.

Name	Consensus length	Total read count	Average coverage
ABT-28_S24_L001_R1_001_24_(paired)_contig_1_mapping	384	155,238	52,800.01
ABT-28_S24_L001_R1_001_24_(paired)_contig_2_mapping	484	24,212	6485.99
ABT-28_S24_L001_R1_001_24_(paired)_contig_3_mapping	253	103	49.93

*Created by CLC De novo Assembler employing CLC Genomics Workbench 12.0

**Table 2:** Consensus sequences of Contig 1, -2 and -3 obtained by *de novo* assembly of NGS reads of phage display library (PDL) of Indian desert camel.

**>PDL-NGS paired Contig 1 sequence (average length-384nt)**
AGTGCGGCCGCTGGAGACGGTGACCTGGGTCCCCTGGCCCCACAATTTATACCCCGTTAAGTCGCCCCAACAATATGGAGTTTCGGT-TGCCGCACAGTAATAGATGGCCGTGTCCTCAGGTTTCAGGCTGTCCATTTGTAGATACACCGTGTTGTTGATGTTGTCTCGGGAGATGG-TGAATCGGCCCTTCACGGACTTGGCGTATACACCGGTTCCCTCAGTATTCACTGATGCGACCCCTTCACGCTCCTTCCCTGGAGCCTGG-CGGACCCAGCCCATACAGTAATTACTGTACGTGTATTTAGGGGCTGCACAGGAGACTCTCAGAGACCCTCCAGTCTGCACCGAGCCTCC-TCCAGACTCCTGCAGCTGCAGGAGCTCTG

**>PDL-NGS paired Contig 2 sequence (average length-484nt)**
TGGGAATCAGAGAAATCGGCACACGGAACATCCTAGGCAGCGTCAGCTGCGGATCAGAGACAGCTGCAGGAGTCTGGAGGAGGCTCG-GTGCAGGCTGGAGGCTCTCTGAAACTCTCCTGTGCAATTTCTGGATACGACAACGATAACTACTGCATGGGCTGGTTCCGCCAAACGC-CAGGGAAGGAGCGTGAGAAAGTCGCGGCCCTTAATATTGGAGGTGGTAGCCCAGTCTACGCCGATTTCGTGAGGGGCCGATTCACCA-TCTCCCTGGACTCCAGCAAGGACACACTGTACCTCCTGATGAACGCAGTGACACCCGAGGACACGGCCATGTATTACTGTGCGGCAATC-CGTAAGCCCCAATTCTATACTTGCCGTATGTGGAAATCAAGAGCTGACTTTGATATCTGGGGCCAGGGGACCCAGGTCCGTCTCCAGC-GGCACAGCGCCGCACCCACGAGACTAGGCATGATCTCGTATGCCG

**>PDL-NGS paired Contig 3 sequence (average length-253nt)**
CAGGAGTCTGGAGGAGGTTCGGTGCAGGCTGGCGGGTCTCTGAGACTCTCTTGCACAACCTCTAGATACATTCCTACAACCAACTGTAT-AGGCTGGTTTCGCCAGGTTCCGGGTAAGGAGCGCGAGAAGGTCGCAGCCCTTCGTACTGGAGATCGTAGTACAACATTTTATGCCGAC-TCCGTCAAGGGCCGATTCACCATCTCCCAAGACGGTGTCAAGAATATAGTGTATCTGGAAATGAACAGCCTGAAAC

IgBLAST (https://www.ncbi.nlm.nih.gov/igblast/igblast.cgi) of contig 1, -2 and -3 consensus sequences revealed human VH subfamily III-like features of camel VHH and identified Framework regions (FR) 1-4 and complementarity determining regions (CDR)1-3 (Mendeley dataset files: IgBLAST_PDL_NGS_Contig1_Search Results; IgBLAST_PDL_NGS_Contig 2_Search Results; and IgBLAST_PDL_NGS_Contig 3_Search Results, respectively [8]). Table 3 shows contig 1 and -2 having CDR1, -2 and -3, whereas contig 3 being of shorter length, having only CDR 1 and -2 (Mendeley dataset file: CDR1-3 in NGS consensus contig 1-3 of PDL_Idc matched with anti-LPS and anti-*T. evansi* VSG Nb clones [8]). In addition, Table 3 shows that the contig 1 CDR1, -2 and -3 are identical to the *T. evansi* VSG Nb clones, which were isolated from the library and sequenced by Sanger's automated technique (Mendeley dataset file: IgBLAST- MW310247 (anti-*T evansi* RoTat1.2 VSG Nb Cl11); IgBLAST- MW310248 (anti-*T evansi* RoTat1.2 VSG Nb Cl17); CDR1-3 in NGS consensus contig 1-3 of PDL_Idc matched with anti-LPS and anti-*T. evansi* VSG Nb clones [8]). The contig 2 CDR 1 and -2 are identical to the LPS-binder Nb clones previously isolated from the library and sequenced by Sanger's automated technique. However, contig 2 CDR3 sequence has only one amino acid position mismatch with four LPS-binders and two amino acid positions with one LPS-binder clone. One randomly picked and Sanger- sequenced clone (EU429319) from the library showed that CDR 1 and -3 were similar to the LPS-binder clones, whereas CDR2 was entirely different. The Table 3 also shows that VHH hallmark amino acids are present in all the contigs, like those in individually isolated clones from the library. Nucleotide blast (‘BLASTn’ at https://blast.ncbi.nlm.nih.gov/Blast.cgi) of contig 1-3 consensus sequences revealed homologies to sequences of Nbs specific to *E. coli* LPS, *T. evansi* variable surface glycoprotein (VSG), foot-and-mouth (FMD) virus serotypes O and Asia-1 capsid protein, porcine circovirus (PCV)-2 antigen, hepatitis C virus (HCV) non-structural (NS)3/4 serine protease, vascular epidermal growth factor (VEGF), and others. (Mendeley dataset file for each contig blast report (NCBI Blast_PDL-NGS Contig 1 (383 nt), NCBI Blast_PDL-NGS Contig 1 (484 nt), and NCBI Blast_PDL-NGS Contig 1 (253 nt), respectively [8]). Further, nucleotide blastn of KF990217.1 (Anti-LPS Nb Cl26 nt1-200 and nt190-384) and like-wise other anti-LPS Nb clones previously selected from the library and aligned to sequence reads archive (SRA) database of the library (SRA-SRX5282797; run SRR8477290) accessible at URL: https://www.ncbi.nlm.nih.gov/Traces/study/?acc=PRJNA516512) (data file ref. [7]) revealed highly similar sequence reads (>95% to >99% with >99% coverage) (Mendeley dataset files: KF990217 (Anti-lps Nb Cl26_nt1-200) versus SRA-SRX5282797 (pdl_ngs reads)- VRZ52J8K01R-Alignment; KF990217 (Anti-lps Nb Cl26_nt190-384) versus SRA-SRX5282797 (pdl_ngs reads)- VRZ52J8K01R-Alignment [8]). Based on homology of consensus contigs to *T. evansi* VSG, we isolated anti-*T. evansi* RoTat1.2 VSG Nb clones by bio-panning of PDL_Idc and Sanger sequenced these clones. Blastn of MW310247 and MW310248 (anti-*T. evansi* VSG Nb Cl11 and Cl17) against SRA database revealed >95% identity with >99% coverage as shown in Mendeley dataset files [8]: Blastn_MW310247 (anti-*T. evansi* Nb Cl11_ nt1-100 versus SRA-SRX5282797 (pdl_ngs reads)-W7BSBJFA01N-Alignment; Blastn_MW310247 (anti-*T. evansi* Nb Cl11_ nt1-200 versus SRA-SRX5282797 (pdl_ngs reads)-W7C0BARS01N-Alignment; Blastn_MW310247 (anti-*T. evansi* Nb Cl11_ nt190-387 versus SRA-SRX5282797 (pdl_ngs reads)-W7CTN7JC01N-Alignment; Blastn_MW310248 (anti-*T. evansi* Nb Cl17_ nt1-100 versus SRA-SRX5282797 (pdl_ngs reads)-W7BFSKY101N-Alignment; Blastn_MW310248 (anti-*T. evansi* Nb Cl17_ nt1-200 versus SRA-SRX5282797 (pdl_ngs reads)- W7AY91YJ01N-Alignment; Blastn_MW310248 (anti-*T. evansi* Nb Cl17_ nt190-384 versus SRA-SRX5282797 (pdl_ngs reads)-W7DHKKKA01N-Alignment. As demonstrated by isolation of anti-*T. evansi* RoTat1.2 VSG Nb clones, the PDL-NGS reads- guided approach could be used for search of Nbs specific to other antigens, and for search of templates suitable for Nb reformatting and Nb-based drug designing.

**Table 3:** Complementary determining region (CDR) 1-3 in consensus contig 1, -2 and -3 of NGS data of the phage display library of Indian desert camel (Identified by IgBLAST at ncbi.nlm.nih.gov/) and those of nanobody clones isolated from the same library and sequenced by automated Sanger's dideoxy chain termination method.

NGS consensus contig#/Sanger-sequenced PDL clone id (GeneBank accession no.)	CDR1	CDR2	CDR3	Camelid VHH Hallmark AA [S11, F37, E44, R45]
Contig 1	KYTYSNYC	VNTEGTG	AATETPYCWGDLTGYKL	Yes, all except V37
Contig 2	GYDNDNYC	LNIGGGSP	AAIRKPQFYTCRMWKSRADFDI	Yes, all
Contig 3	RYIPTTNC	LRTGDRSTT	—–MISSING—-(SHORT SIZE)	Yes, all
Anti-LPS Nb Cl26 (KF990217)	GYDNDNYC	LNIGGGSP	AAIRKPRFYTCRMWKPRADFDI	Yes, all
Anti-LPS Nb Cl23 (KF990216)	GYDNDNYC	LNIGGGSP	AAIRKPQFYTCRMWKPRADFDI	Yes, all
Anti-LPS Nb Cl16 (KF990215)	GYDNDNYC	LNIGGGSP	AAIRKPQFYTCRMWKPRADFDI	Yes, all
Anti-LPS Nb Cl18 (KM885986)	GYDNDNYC	LNIGGGSP	AAIRKPQLYTCRMWKPRADFDI	Yes, all
Anti-LPS Nb (EU861212)	GYDNDNYC	LNIGGGSP	AAIRKPQFYTCRMWKPRADFDI	Yes, all
EU429319 (Unknown specificity)	GYDNDNYC	VSGLSGVSKTT	AAIRKPQFYTCRMWKPRADFDI	Yes, all
Anti-*Trypanosoma evansi* RoTat1.2 VSG Nb Cl11 (MW310247)	KYTYSNYC	VNTEGTG	AATETPYCWGDLTGYKL	Yes, all except V37
Anti-*T. evansi* RoTat1.2 VSG Nb Cl17 (MW310248)	KYTYSNYC	VNTEGTG	AATETPYCWGDLTGYKL	Yes, all except V37

**Fig. 3:**
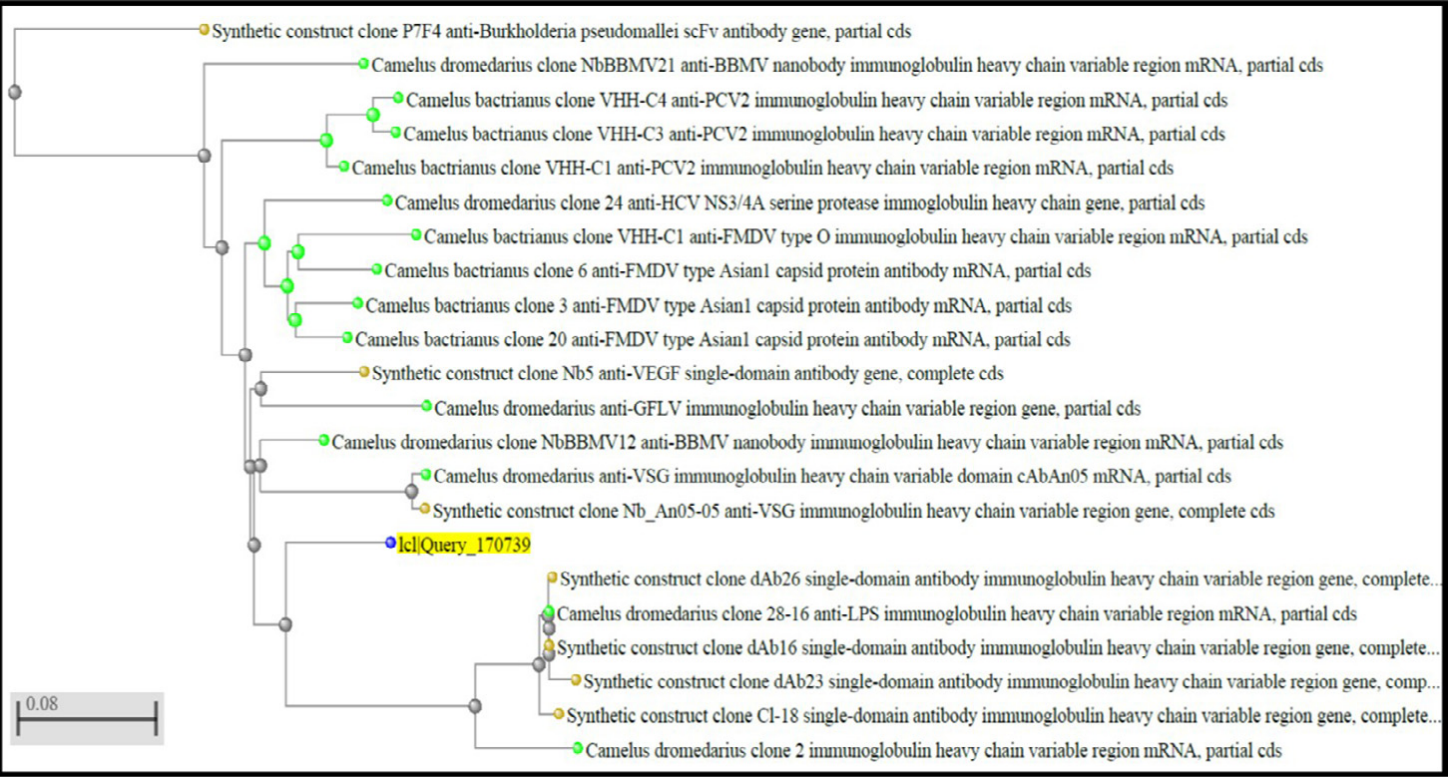
Phylogenetic tree (Neighbor-Joining) of contig 2 consensus sequence aligned to selected Nb clones using ‘BLASTn’ at NCBI.

Fig. 3 presents the phylogenetic tree showing relatedness of contig 2 consensus sequence to various nanobodies. The homology-based evidence led us to select successfully *T. evansi* Ag-specific Nb clones by panning of our phage display library. Like-wise, we are attempting to isolate Nb clones that bind Ags from veterinary pathogens such as *Pasteurella multocida* B:2 LPS, FMD virus serotypes, *Staphylococcus aureus* exotoxins, etc. As mentioned above, the homology-based evidence also exists for various Nbs against other animal and human pathogens, and VEGF, thereby strengthening our notion that the phage display library in its existing form is a valuable Nb resource of Indian origin.

## 2 Experimental Design, Materials and Methods

### 2.1 The phage display library of nanobodies derived from Indian desert camel: the background information

The present NGS dataset was obtained from a previously constructed and cryo-preserved Nb-phage display library derived from Indian desert camel (PDL-Idc). The PDL-Idc was constructed according to Ghahroudi et al. [4], with some modifications in our laboratory during 2006-07. Standard rDNA and molecular cloning protocols had been followed [5]. The 370–420 bp Nb PCR amplicon pool was directionally cloned into *PstI* and *NotI* sites of pHEN4, the phage display vector, to allow expression of Nb with C-terminal decapeptide hemagglutinin (HA) tag in fusion with M13 phage pIII minor coat protein in the transformed amber-suppressor *E. coli* strain TG1. With transformation efficiency of about 80%, the size of Nb-pHEN4 transformant TG1 library was estimated to be comprising of >5 × 10^7^ Nb clones. The TG1 library clones were cryopreserved in LB/amp/glu/30% glycerol at –70 °C. When required, the library of Nb-displaying phages was rescued by infection of the above TG1 transformants with M13K07 helper phage. From the phage library, *E. coli* LPS-binders and *S. aureus* β-hemolysin-binder Nb-displaying phage clones were selected by panning following a standard protocol, expressed and characterized [6]. Subsequently, a few Nb clones of LPS-binders and *S. aureus* hlb-binders were expressed, purified, characterized and sequenced by Sanger's automated technique. Nucleotide sequences of representative Nb clones were submitted to NCBI GenBank [http://www.ncbi.nlm.nih.gov/BankIt/]. Immuno- and bioinformatics techniques revealed that these sequences (GenBank Accession no. EU429319, EU861212, KF990215; KF990216; KF990217; GU014816) had the camelid VHH hallmark amino acids, i.e., S11, F37, E44, R45, and were phylogenetically closer to the Arabian camel VHH sequences than to llama and other new world camelids.

### 2.2 Extraction of VHH-pHEN4 phagemid DNA from TG1 transformant library for NGS

The transformant library was revived by thawing at room temperature (RT). The library culture aliquot (1.5 mL) was inoculated in 150 mL of LB/Amp broth in sterilized conical flasks and grown at 37 °C with shaking for 20 h [6]. VHH-pHEN4 phagemid DNA was extracted from 100 mL of the above culture of the TG1 transformants library and purified using commercially available maxi-column (Qiagen, Germany) according to the manufacturer's protocol. The purified DNA was quantified by A_260_, and its purity assessed by agarose gel electrophoresis (AGE) and A_260_/A_280_ ratio.

### 2.3 VHH-PCR of Nb clones

VHH-PCR was performed for amplification of VHH clones (that encode for Nb clones) using VHH-specific primers, viz., A6E and FR4For [6]. Ten reactions, each of a final volume of 50 μL were set-up and carried out in a thermocycler as follows: The reaction components were added in sequence as follows: nuclease-free water, 2x PCR buffer master mix (Dream Taq Green master mix, Thermo Scientific), 200 pM each primer and 5.0 μL template DNA (VHH-pHEN4 purified above), making the final reaction volume 50 μL in 0.2 mL PCR tube. The DNA was amplified in ABI thermocycler using protocol: 94 °C for 4 min, 30 cycles of [denaturation at 94 °C for 1 min, annealing at 63 °C for 1 min and extension at 72 °C for 1 min], final extension at 72 °C for 10 min. and held at 4 °C. The PCR product was stored at –20 °C till further use.

### 2.4 Agarose gel electrophoresis (AGE) of PCR products for extraction and purification

PCR products were resolved by AGE in 2% agarose in 1X tris-acetate-EDTA buffer, pH 8.3 (1x TAE) in a mini-gel electrophoresis apparatus at 5 V/cm of gel length [5]. A 100 bp DNA ladder (Thermo Scientific, USA) was run for determining the size of PCR products. The VHH-PCR products resolved by AGE above were visualized at 312 nm in a gel documentation system and the 370–420 bp bands excised [5]. The gel pieces were transferred to 1.5 mL Eppendorf microfuge tubes, and DNA extracted and purified using a commercial gel extraction kit (Qiagen, Germany). The DNA was eluted in 10 mM tris-HCl, pH 8.5 and stored at –20 °C till further use. DNA was quantified by A_260_ and the purity assessed by A_260_/A_280_ ratio.

### 2.5 NGS of the library of Nb clones by Illumina® MiSeq™ system

DNA library of Nb sequences was subjected to Illumina® MiSeq™ system available in the Department of Animal Biotechnology, LUVAS, Hisar. The NGS data of the library DNA sample was acquired and initially analyzed with the software available on the platform. ‘Nextra XT micro V2 Index’ kit (with total output data of 800 Mb and 8 million paired-end reads of 150 bp length) was used for the Nb library DNA sample sequencing, with following adaptors: ‘i7’ (N706: TAGGCATG) and ‘i5’ (S517: GCGTAAGA). Raw NGS fastq data files were deposited at NCBI Sequence Reads Archive (SRA). The SRA data with Accession no.PRJNA516512 was released for public access on 22 February 2020, and the run SRR8477290 are accessible at URL: https://www.ncbi.nlm.nih.gov/Traces/study/?acc=PRJNA516512 (dataset ref. [7]). After preliminary analysis, the dataset was deposited at Mendeley Datasets Repository, and version 3, which is accessible at URL: [https://data.mendeley.com/datasets/4rsz3snvk5/3] was published on 08 December 2020 (dataset ref. [8]).

### 2.6 Bioinformatics for determination of genetic diversity of the nanobodies library

Quality scores of the raw NGS reads, trimming of adaptors, etc. was done by the software of MiSeq™ system. Contigs to get full-length nucleotide sequence of Nb clones was done using De Novo Assembler at CLC Genomics Workbench 12.0 (Qiagen Bioinformatics). Basic molecular biology tools available at NCBI and CLC software were also used. Copy numbers of the sequence reads in the library were estimated. Searches within the databases were done using NCBI Nucleotide Blast [URL: https://blast.ncbi.nlm.nih.gov/Blast.cgi] and IgBLAST [URL:https://www.ncbi.nlm.nih.gov/igblast/igblast.cgi] [9], [10], [11], [12]. The dataset analysis files are available at Mendeley dataset repository (dataset ref. [8]).

## Ethics Statement

The work to obtain and analyse NGS dataset of the phage display library did not involve the use of humans or animals.

## CRediT Author Statement

Somesh Banerjee: Methodology, investigation; Ajit Singh.: Conceptualization, supervision, data analysis; Writing- Original draft preparation; Jagveer Rawat: investigation, supervision, Sushila Maan: Methodology, data analysis; Nitish Bansal: Methodology, data analysis.

## Declaration of Competing Interest

The phage display library of nanobodies derived from LPS-immunized Indian desert camel was produced in 2009 in a research project funded by Department of Biotechnology (DBT), Government of India and implemented by the second author as Principal Investigator at Ch. Charan Singh Haryana Agricultural University (CCSHAU), Hisar, Haryana. The said library is an Nb resource of implied commercial interests and is a joint intellectual property of the funding agency (DBT) and CCSHAU according to mutually agreed terms and conditions. NGS of Nbs in the said library has been funded by ICAR, New Delhi for Emeritus Scientist scheme being implemented in LUVAS, Hisar. Any commercial interests arising from these findings will be jointly settled by LUVAS, Hisar and ICAR, New Delhi. However, these declared ‘Conflicts of Interest’ did not influence the results of the present investigation.
